# Evolution and trends in tremor diagnosis and differential diagnosis—a bibliometric study

**DOI:** 10.3389/fnagi.2026.1829580

**Published:** 2026-07-27

**Authors:** Jiayun Liu, Xiangming Xu, Jingliang Liu, Ruihao Bian, Jiaxuan Zheng, Ke Dong, Xiaoxia Zhu, Xi Chen

**Affiliations:** 1 Department of Rehabilitation Medicine, The First Affiliated Hospital of Sun Yat-sen University, Guangzhou, Guangdong, China; 2 Department of Rehabilitation Medicine, Zhongshan Torch Development Zone People's Hospital, Zhongshan, Guangdong, China

**Keywords:** bibliometric analysis, diagnosis, differentiation, movement disorders, tremor

## Abstract

**Background:**

Tremor is one of the most common motor symptom across movement disorders, and its precise diagnosis has become increasingly critical. However, existing studies remain fragmented. They often focus on specific disease manifestations or isolated diagnostic techniques, but lack systematic analysis. A comprehensive review is therefore needed to consolidate insights and guide future diagnostic advancements.

**Methods:**

We systematically searched the Web of Science Core Collection (WoSCC) and ClinicalTrials.gov (January 2016–August 2025), identifying 1,145 publications and 47 trials. Bibliometric networks were then visualized and analyzed using CiteSpace, VOSviewer, and the R package “bibliometrix.”

**Results:**

Annual publication volume increased steadily, while citations rose from 2016 and peaked in 2021. The United States led in publications, whereas Germany held the highest betweenness centrality. Yale University ranked first in publication output, and University College London had the greatest betweenness centrality. Louis, Elan D was the most influential author, leading in publication count, citation frequency, and betweenness centrality. *Parkinsonism and Related Disorders* was the most productive journal. Clinical trials predominantly focused on essential tremor (ET) and majorly employed device-based methodologies. Research centered on four themes including quantitative tremor assessment and digital health technologies, neurophysiological evaluation, neuroimaging, and pathophysiology and biomarkers, with machine learning (ML) and deep learning (DL) emerging as key frontiers.

**Conclusion:**

This study comprehensively maps the research landscape in tremor diagnosis and differential diagnosis over the past decade. On this basis, it identifies current limitations, and predicts emerging research frontiers, thereby offering valuable insights and guidance for future scientific investigation.

## 1 Introduction

Tremor, defined as an involuntary rhythmic, oscillating movement of a body part (De et al., 2023), is one of the most common motor symptom across various movement disorders, including Parkinson’s disease (PD), atypical parkinsonian syndromes, essential tremor (ET), and dystonia. According to the International Parkinson and Movement Disorder Society (IPMDS), PD is characterized by bradykinesia in combination with resting tremor or rigidity. ET is defined as an isolated tremor syndrome with bilateral postural or kinetic tremors lasting at least 3 years, with or without tremor in other body regions. Collectively, these indicate the central role of tremor in the diagnosis of movement disorders.

A refined tremor taxonomy has further driven research on tremor phenomenology and etiology. In 2018, the IPMDS updated the consensus criteria for tremor classification and proposed a dual-axis framework: Axis 1 based on clinical phenomenology (tremor syndrome) and Axis 2 based on etiology (Bhatia et al., 2018). This refined classification has promoted more precise diagnosis and differentiation of tremor syndromes. Over the past decade, research on tremor has expanded substantially across neurophysiology (Chen and Chen, 2020), neuroimaging (Buijink et al., 2022), molecular biology (Barbagallo et al., 2017), and behavioral assessments such as voice and drawing analysis (Snow and Guardiani, 2019; Anandapadmanabhan et al., 2025). The field has progressed from an initial focus on differentiating Parkinson’s tremor (PT) and ET to investigations of underlying mechanisms and, more recently, the integration of digital health technologies such as artificial intelligence (AI) and wearable sensors (Anandapadmanabhan et al., 2025; Dattola et al., 2025).

Despite these advances, existing studies have largely focused on tremor associated with specific diseases or particular diagnostic modalities (Zhang et al., 2024). Therefore, a comprehensive analysis covering various diagnostic approaches for different tremor types remains lacking. Bibliometric methods can synthesize large volumes of literature, visualize collaboration networks, and elucidate knowledge evolution, thereby systematically tracking evolving research trends and emerging hotspots to guide future advances in tremor diagnosis and differential diagnosis (Chen, 2004). Additionally, while previous studies have seldom incorporated evidence from clinical trials, clinical trials analysis can not only reveal the true technological value that is amenable to clinical translation, but also show the current status and future directions of clinical research in this field. In this study, we integrated relevant publications from 2016 to August 31, 2025, and conducted a comprehensive bibliometric analysis using established bibliometric tools. Specifically, we outlined the general research landscape, identified limitations, and predicted future frontiers to offer insights for scholars in this field.

## 2 Method

### 2.1 Data sources and search strategy

Bibliometric data were retrieved from the Web of Science Core Collection (WoSCC), spanning publications from January 2016 to August 31, 2025. Since bibliometric tools do not have built-in machine translation or cross-language synonym dictionaries, the language was restricted to English. The search strategy utilized the following query: TS = (tremor OR tremors) AND TS = (screening OR detection OR diagnosis OR assessment OR predict OR estimate OR identify OR measure OR biomarker OR biomarkers OR classify OR classification OR types OR phenotypes OR differential OR differentiation OR quantification). The initial search yielded 10,243 publications. After excluding non-peer-reviewed document including proceeding papers, news, information, early accesses, meeting abstracts, editorial materials, retracted publications, letters, book chapters, data papers, corrections, meeting and reprint, 9,059 articles were retained. Subsequent manual screening based on the inclusion criteria identified 1,145 eligible publications.

### 2.2 Study selection and data collection

**Figure 1:**
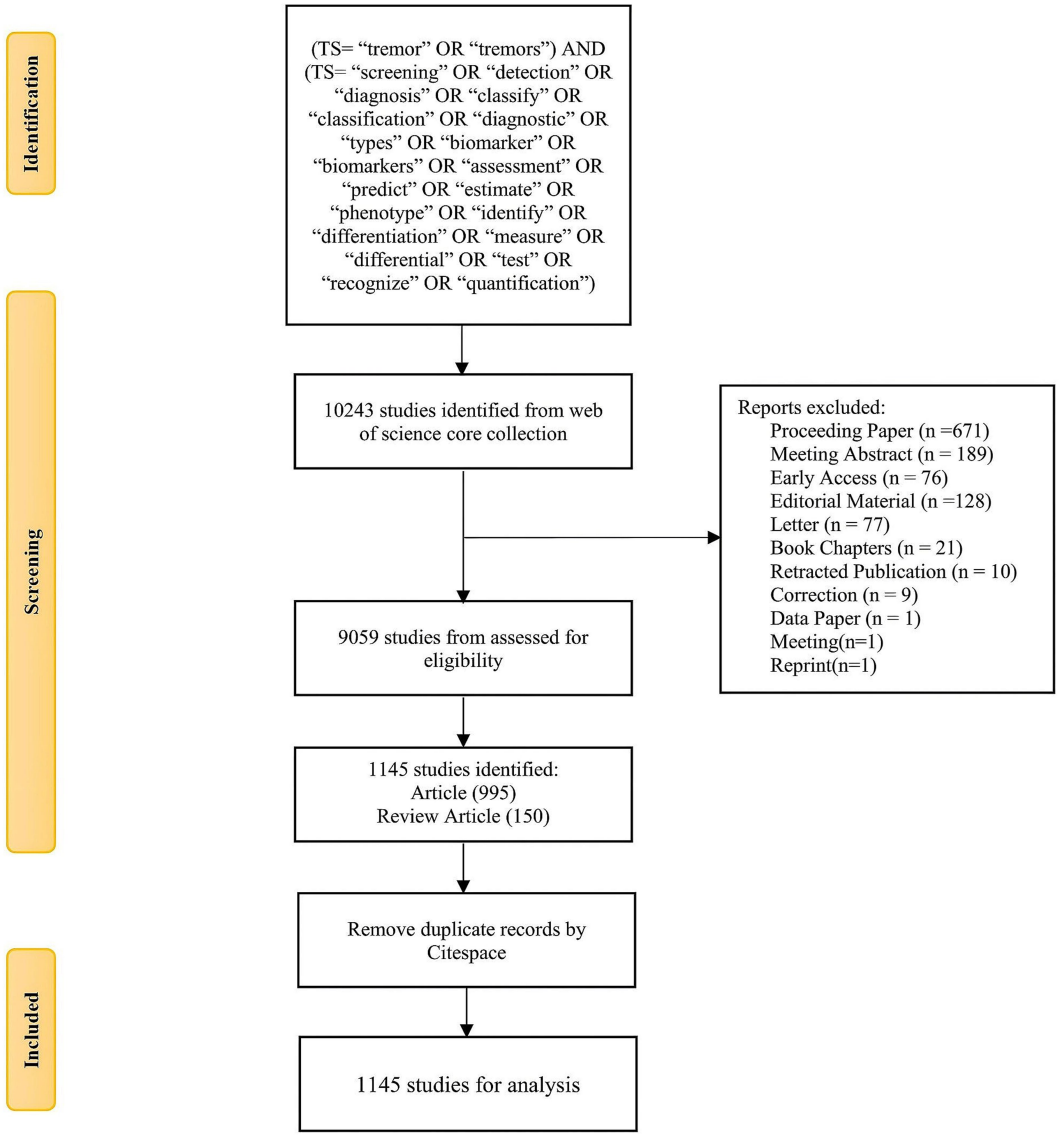
The flowchart of the literature selecting process.

The screening of the 9,059 records was performed independently by two investigators who evaluated each article based on title, abstract, and keywords against the inclusion criteria. To mitigate the impact of database updates, data collection was completed on August 31, 2025. The inclusion criteria were: (1) articles or reviews focusing on the diagnosis and differential diagnosis of tremor; (2) studies containing accessible bibliometric metadata, including title, authors, affiliations, abstract, keywords, and citation counts. The exclusion criterion was duplicate publications. The two reviewers compared their screening results and retained the items on which they agreed; disagreements were resolved through discussion with a third reviewer. Finally, a total of 1,145 eligible publications were identified. The selected articles were exported in plain text format, archived in a dedicated folder, and analyzed for bibliometric data—including titles, authors, affiliations, journals, keywords, references, and related citation information. The search workflow was illustrated in Figure 1.

### 2.3 Bibliometric analysis

CiteSpace 6.4. R1, VOSviewer and R package “bibliometrix” were applied to visualize bibliometric networks, facilitating an in-depth analysis of relevant research. In CiteSpace, time span was set from 2016 to 2025, with annual slices. The cosine algorithm was employed to calculate link strength between network nodes. The node types selected were as follows: countries, institutions, authors, journals, and keywords, which were used to generate collaboration networks, a dual-overlay map, a timezone view of keywords, and citation bursts. For collaboration networks (countries, authors, institutions), a threshold of the top 20 most frequent items per slice was applied, along with the keyword co-occurrence analysis was limited to the top 50 terms per slice. Network optimization was performed using Pathfinder and Pruning Sliced Networks algorithms. The default value were used for other parameters. In the resulting visualizations, node size corresponds to publication volume or keyword co-occurrence frequency, line width represents collaboration intensity, and colored rings denote the temporal distribution across years. Purple outer rings highlight nodes with high betweenness centrality (≥0.1) (Synnestvedt et al., 2005). As for VOSviewer, the default value were used for all parameters. Co-authorship networks were constructed with a threshold of minimum 5 documents per author, and keyword co-occurrence networks required at least 15 occurrences. Node size represents the total link strength of co-authorship relationships or keyword co-occurrence frequency, connecting lines depict collaboration strength, and distinct colors represent different thematic or collaborative clusters. In R package “bibliometrix,” the core journals were identified based on Bradford’s Law.

### 2.4 Clinical trials

Clinical trial data were extracted from ClinicalTrials.gov using the search query “tremor OR tremors.” The study date was set from January 1, 2016, to August 31, 2025. No filters were imposed on study status, eligibility criteria, study phase, study results, or study documents. This search strategy identified a total of 256 clinical trials. After excluding trials unrelated to the diagnostic and differential diagnosis of tremor, 47 studies were selected. In this manual screening process, all data were independently extracted by two authors and cross-verified. Finally, data were exported from the ClinicalTrials.gov website and then were systematically extracted by Excel spreadsheets for the following variables: NCT identifier, study title, study status, study type, start date, interventions, primary outcome measures, primary completion date, sponsor, study locations, and collaborating institutions. These data were subsequently synthesized to deliver a comprehensive overview of clinical trial activity in this field over the past decade.

## 3 Result

### 3.1 Publication volume and citation trends

**Figure 2:**
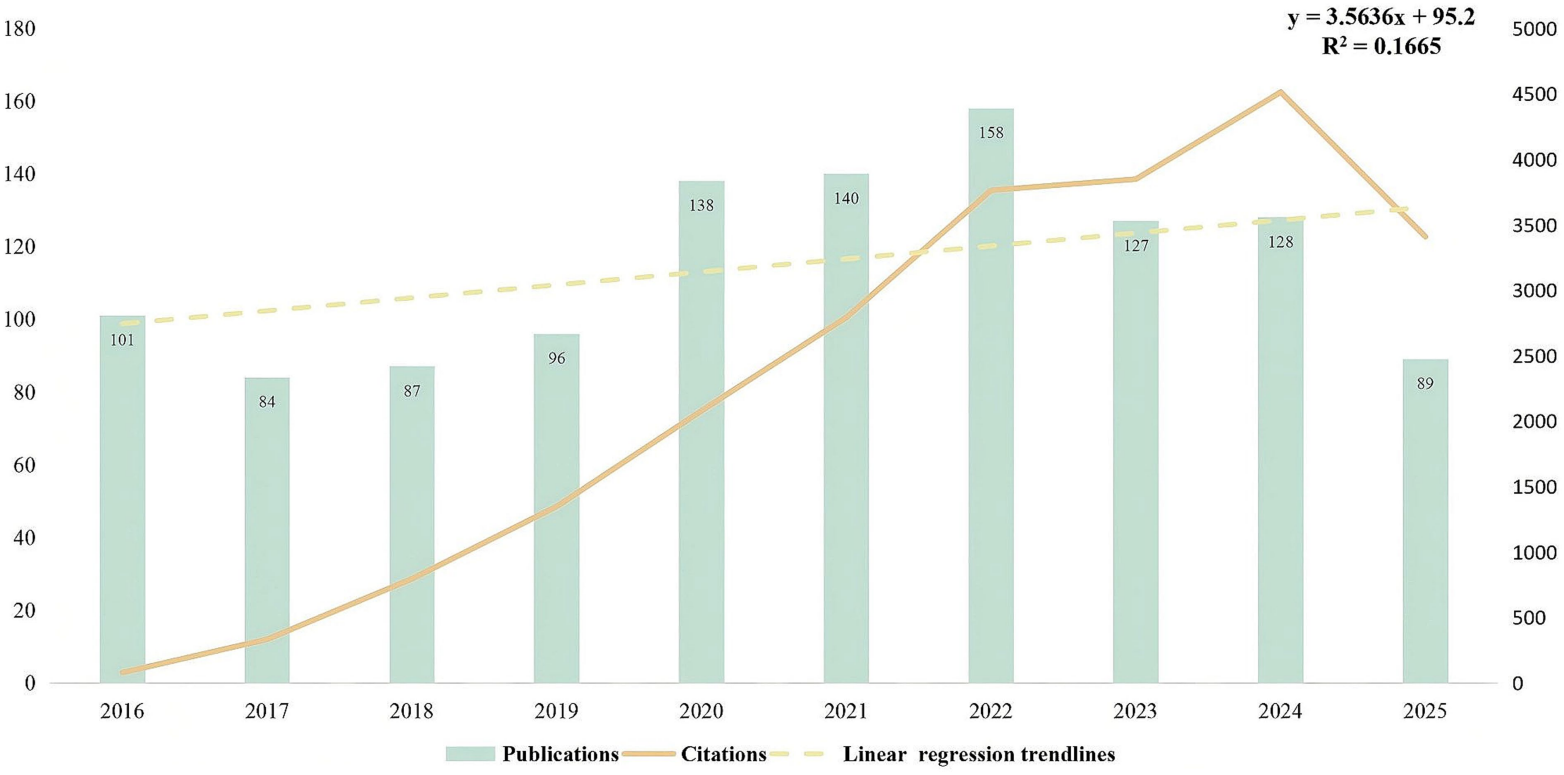
The number of publications and citations annually. The green bar chart displays the annual publication count with its linear regression trend shown as a dashed line, and the yellow line represents the annual citation counts.

A total of 1,145 publications focusing on tremor diagnosis and differential diagnosis were identified in the WOSCC database between 2016 and 2025. On average, there were 115 publications annually over the first 9 years, with 89 additional publications published by the end of August 2025. As shown in Figure 2, the green bar chart displays the annual number of publications, while the dashed line represents the linear regression trend. This model reveals a significant and steady increase in publication output, with an average annual growth of approximately 13.4 publications. Notably, there was a sustained growth phase from 2017 to 2022, during which publications increased by an average of 14.8 articles per year. The peak publication count was observed in 2022, reaching 158 articles, indicating continuing activity in this research domain during this period.

The research domain involved 5,581 authors from 1,201 institutions across 201 countries/regions, who published their work in 424 academic journals. The 1,145 included publications accumulated 22,884 citations, yielding a mean of 19.92 citations per article. The field achieved an H-index of 64, indicating a mature research ecosystem with substantial academic impact. In Figure 2, the yellow line displays the annual citation counts, demonstrating a consistent upward trend from 2016 to 2024. This trend reflects the growing influence of publications in this domain over the past decade, highlighting persistent scientific interest and continued growth momentum.

### 3.2 Productivity and collaboration network of country and institution

**Figure 3:**
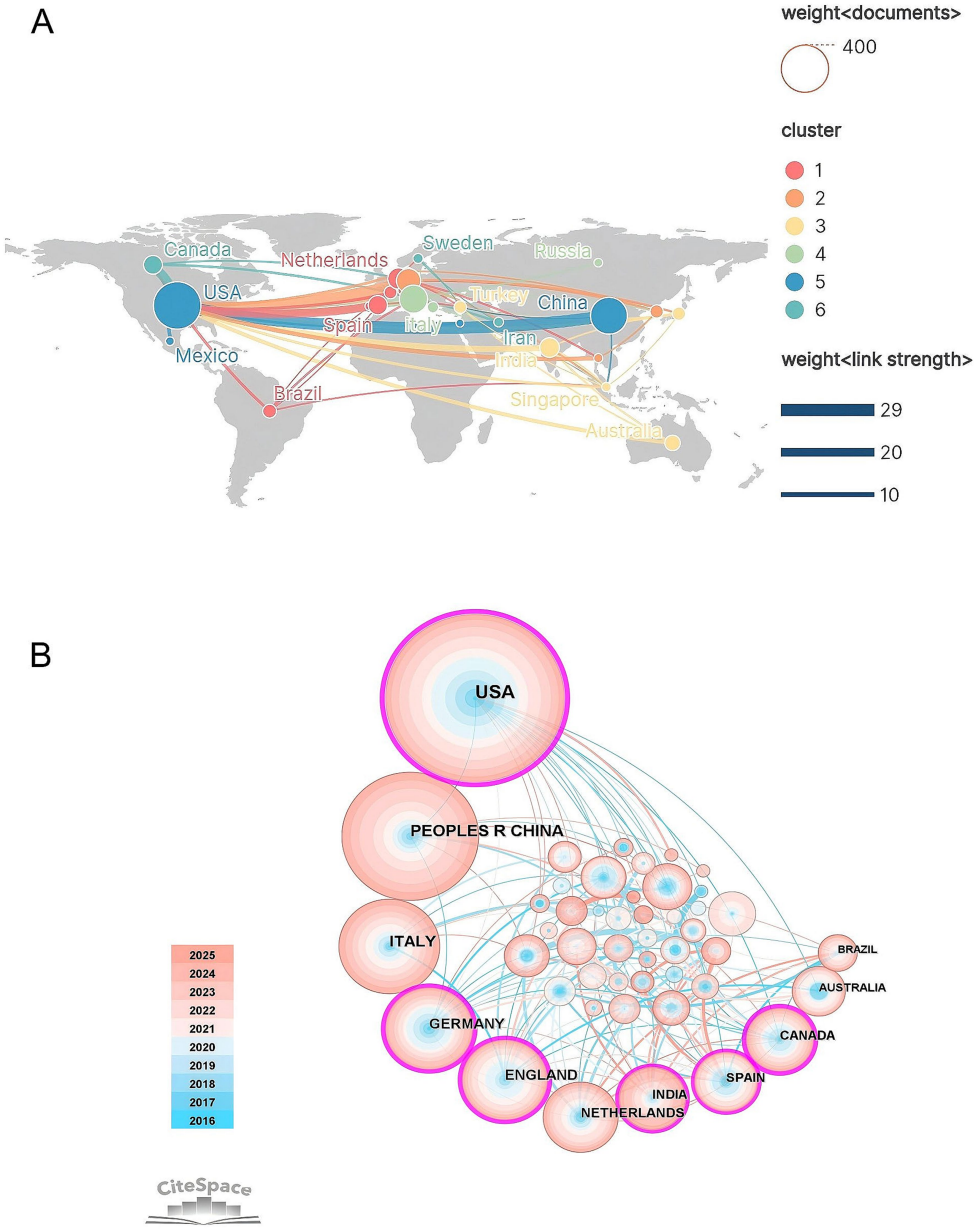
Analysis of countries/regions. **(A)** National Collaborative Network. Only countries with more than 10 publications are displayed in the map. Node size corresponds to publication volume, line width corresponds to collaboration intensity among countries/regions, and countries/regions with close collaborations are assigned the same color. **(B)** Country Co-authorship Network Map. Node size, line width, and colored rings encode publication volume/keyword frequency, collaboration intensity, and temporal distribution, respectively. Purple outer rings denote nodes with elevated betweenness centrality (≥0.1).

Tables 1, 2 summarize the leading 10 countries and top 5 institutions ranked by publication volume and betweenness centrality. The United States led the ranking with 363 publications, accounting for 31.53% of total output, followed by China (*n* = 217, 18.95%), Italy (*n* = 127, 11.09%), Germany (*n* = 94, 8.10%), and England (*n* = 93, 8.12%). Germany demonstrated the highest betweenness centrality, followed by England, Canada, the United States, and Italy, reflecting their extensive collaborative networks and pivotal roles as hubs in international collaboration. Figure 3A shows that the national collaborative network was divided into six clusters. Cluster 1 (pink) was predominantly located in Europe, including Spain, France, Germany, and the Netherlands. Cluster 2 (orange) exhibited a broad geographical spread, encompassing South Korea, Germany, and Thailand. Cluster 3 (yellow) was concentrated in Asia, including Singapore, India, and Japan, with Australia also participating. Cluster 4 (green) was primarily clustered around Southern Europe and Russia. Cluster 5 (blue) was the largest cluster and was centered on the two countries with the highest publication output—China and the United States. Cluster 6 (cyan) was a relatively small cluster, represented by Canada and Sweden. Among these, the collaboration between China and the United States was the strongest, followed by collaborations between the United States and Germany. Notably, aside from Cluster 3 and Cluster 2, inter-country collaborations were generally dispersed, which shows distinct regional characteristics. As illustrated in Figure 3B, the United States recorded the highest publication output and betweenness centrality, establishing it as a central hub in international collaborative networks within this research field.

**Table 1:** Top 10 countries and institutions in number of publications.

Field	TOP10	Counts
Countries/Regions	USA	361
	China	217
	Italy	127
	Germany	94
	England	93
	Netherlands	72
	India	62
	Spain	59
	Canada	56
	Australia	39
Institutions	Yale University	24
	Columbia University	22
	University College London	22
	Magna Graecia University of Catanzaro	21
	Radboud University Nijmegen	18
	NINDS	18
	University of Kiel	18
	Sapienza University Rome	18
	Zhejiang University	17
	Capital Medication University	16

**Table 2:** Top 5 countries and institutions in centrality.

Field	TOP5	Centrality
Countries/Regions	USA	0.59
	Germany	0.24
	India	0.22
	Canada	0.16
	England	0.16
Institutions	University College London	0.31
	University of Kiel	0.17
	Columbia University	0.15
	Radboud University	0.13
	Imperial College London	0.12
	CIBERNED	0.11

**Figure 4:**
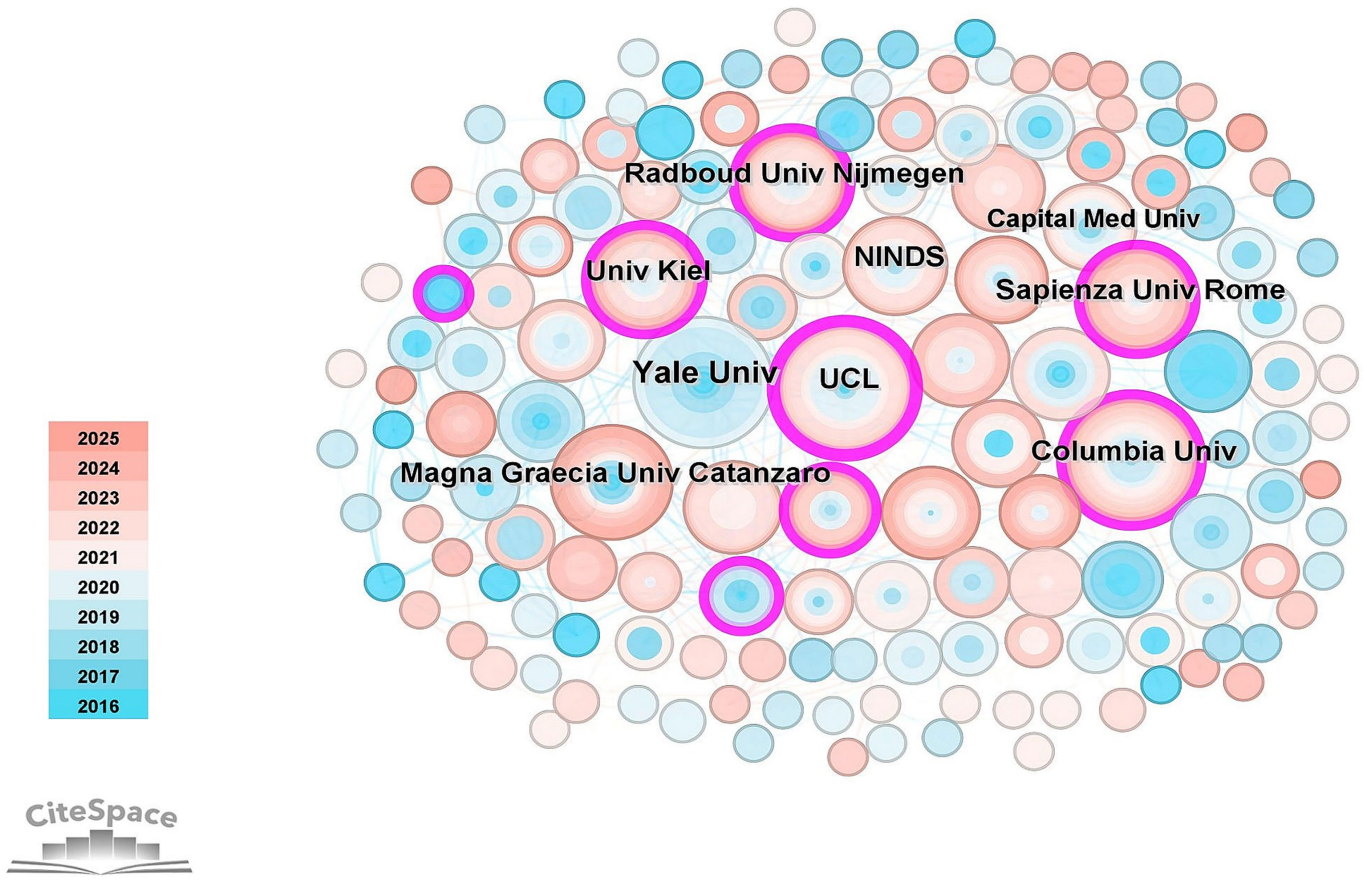
Institutional collaboration map. Node size indicates publication volume, line width indicates collaboration intensity among institutions, and colored rings indicate temporal distribution across years. Nodes with high betweenness centrality (≥0.1) are marked by a purple outer ring.

Among institutions, Yale University recorded the highest number of publications (*n* = 24), followed by Columbia University (*n* = 22), University College London (*n* = 22), and Magna Graecia University of Catanzaro (*n* = 21). The top five institutions ranked by betweenness centrality were University College London, University of Kiel, Columbia University, Radboud University Nijmegen, and Imperial College London. As shown in map of institutions, Columbia University, University College London and Radboud University Nijmegen demonstrated strong performance in both publication volume and centrality metrics, indicating that inter-institutional collaboration predominantly revolves around these three hubs (Figure 4).

### 3.3 Productivity and collaboration network of author

**Figure 5:**
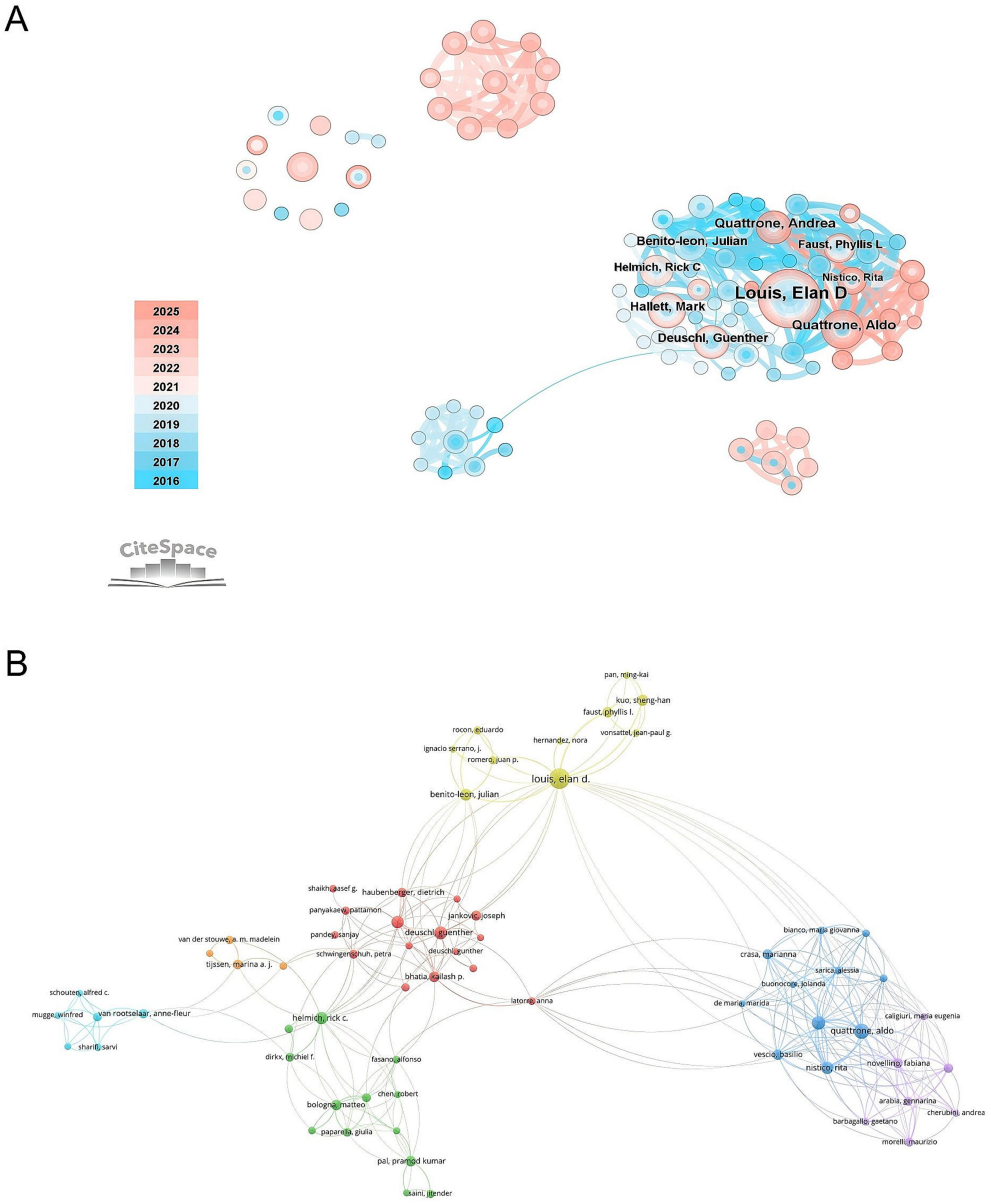
Author co-authorship network map. **(A)** CiteSpace network map. Publication volume, collaboration intensity, and temporal distribution are encoded by node size, line width, and colored rings, respectively. Purple outer rings denote nodes exhibiting high betweenness centrality (≥0.1). **(B)** VOSviewer co-authorship map. Authors are shown based on a threshold of more than 5 publications. Node size represents the total co-authorship link strength, connecting lines represent collaboration strength, and distinct colors represent different thematic or collaborative clusters.

Table 3 presents the top 5 authors ranked by publication count, betweenness centrality, and total citations. The most prolific author was Louis, Elan D (*n* = 44), followed by Quattrone Aldo (*n* = 20), Quattrone Andrea (*n* = 16), Hallet Mark (*n* = 15), and Deuschl Guenther (*n* = 14). Notably, the most productive author contributed only 3.8% of total publications. This distribution suggests a highly decentralized nature without dominance by a small core of scholars. This structural nature is further supported by the author collaboration network generated by CiteSpace (Figure 5A). The maximum betweenness centrality among nodes remained below 0.1, pointing to a decentralized pattern of collaboration. This decentralized pattern likely arises from the interdisciplinary nature of tremor research and its dependence on multicenter, cross-team collaborations, thereby promoting diversified scholarly inquiry and innovation.

**Table 3:** TOP 5 authors in number of publications, centrality and number of total citations.

Publications		Centrality		Total Citations	
Authors	Counts	Authors	Counts	Authors	Counts
Louis ED	44	Louis ED	0.06	Louis ED	389
Quattrone Aldo	20	Bahatia Kailash P	0.05	Deuschl Guenther	338
Quattrone Andrea	16	Benito-leon Julian	0.03	Bahatia Kailash P	271
Hallet Mark	15	Deuschl Guenther	0.02	Jankovic, joseph	235
Deuschl Guenther	14	Van rootselaar Anne-Fleur	0.02	Goetz, Christopher	225

The five most cited authors were Louis, Elan D (*n* = 389), Deuschl Guenther (*n* = 338), Bahatia Kailash P (*n* = 271), Jankovic, Joseph (*n* = 235), and Goetz, Christopher (*n* = 225), demonstrating their considerable academic influence. Notably, Louis, Elan D maintained leading positions across multiple bibliometric indicators, including publication count, betweenness centrality, and citation frequency, highlighting his central and influential role in this research network over the past decade.

VOSviewer co-authorship map (Figure 5B) reveals that the research community can be categorized into seven major collaborative clusters. The largest cluster centered on Quattrone Aldo, while Louis, Elan D maintained the most extensive cross-cluster collaborations. The remaining clusters demonstrated predominantly intra-cluster collaborations. Despite Quattrone Aldo being the largest research cluster, Louis, Elan D demonstrated the strongest total connectivity within the collaboration network, as reflected by node size, confirming his central role in this research domain.

### 3.4 Productivity and dual-map overlay of journal

**Figure 6:**
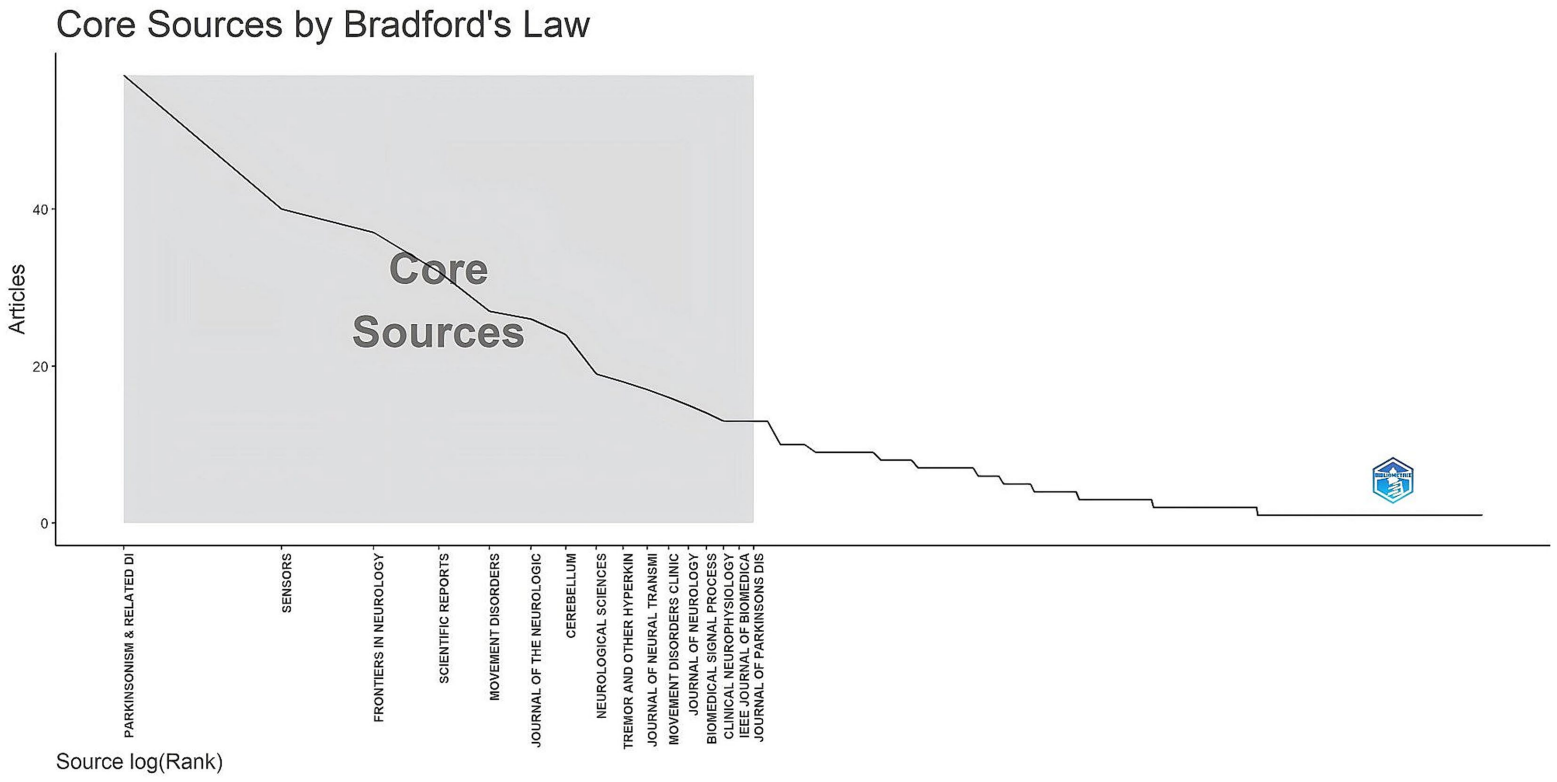
Core journals identified by Bradford’s Law.

Publications in the tremor research domain were distributed across leading journals spanning fields such as movement disorders and neuroscience. According to Bradford’s Law, the majority of significant publications in a field were concentrated in a small number of journals (Espinoza-Carhuancho et al., 2025). This distribution of publications typically follows a pattern of 1: a: a^2^ across core, related, and peripheral journal zones, where the Bradford multiplier (a) approximates 5. The analysis based on Bradford’s Law performed by the R package “bibliometrix” indicated that the top 16 journals by publication output belongs to core journals within this field (Figure 6), including *Parkinson & Related Disorders* (*n* = 57, 4.96%), *Sensors* (*n* = 40, 3.48%), *Frontiers in Neurology* (*n* = 37, 3.22%), *Scientific Reports* (*n* = 32, 2.79%), *Movement Disorders* (*n* = 27, 2.35%). As shown in Table 4, core journals were primarily classified as Q1/Q2 journals, reflecting the high quality of publications in this field and demonstrating the establishment of a stable research framework that balances both quality and quantity.

**Table 4:** The number of publications, IF, and JCR quartile of the core journals identified by Bradford’s Law.

Journals	Counts	Percentage	IF (2024)	JCR	Zone
Parkinson and Related Disorders	57	4.978%	3.4	Q2	core
Sensors	40	3.493%	3.5	Q2	core
Frontiers in Neurology	37	3.231%	2.8	Q2/Q3	core
Scientific Reports	32	2.795%	3.9	Q1	core
Movement Disorders	27	2.358%	7.6	Q1	core
Journal of the Neurological Sciences	26	2.271%	3.2	Q2	core
Cerebellum	24	2.096%	2.4	Q3	core
Neurological Sciences	19	1.659%	2.4	Q3	core
Tremor and other Hyperkinetic Movements	18	1.572%	2.1	Q3	core
Journal of Neural Transmission	17	1.485%	4.0	Q1 /Q2	core
Movement Disorders Clinical Practice	16	1.397%	2.7	Q2	core
Journal of Neurology	15	1.310%	4.6	Q1	core
Biomedical Signal Processing and Control	14	1.223%	4.9	Q2	core
Clinical Neurophysiology	13	1.135%	3.6	Q1/Q2	core
IEEE Journal of Biomedical and Health Informatics	13	1.135%	6.8	Q1	core
Journal of Parkinsons Disease	13	1.135%	5.0	Q1	core

**Figure 7:**
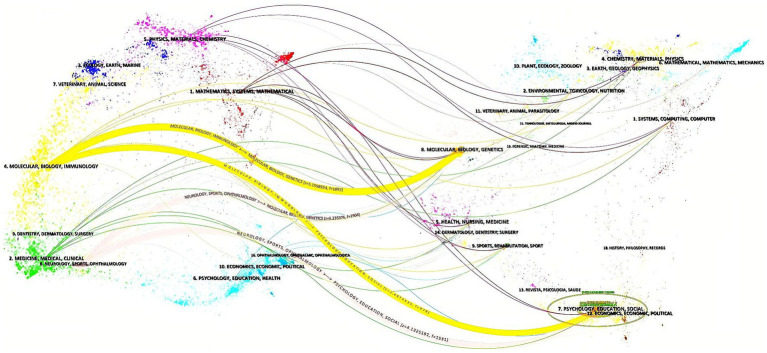
Dual-Overlay Map for Journals. The colored trajectories connecting the two domains represent citation flows, with line widths proportional to citation frequency.

Figure 7 presents a dual-map overlay of journal citations, where cited journals are positioned on the right and citing journals on the left. Analysis of the map indicates that knowledge development in this field followed a crossover pattern. Research on tremor diagnosis and differential diagnosis primarily based on molecular biology, genetics, psychology, and social sciences, with these knowledge elements being subsequently applied and further developed in molecular biology, immunology, neurology, kinesiology, and ophthalmology.

### 3.5 Co-occurrence analysis and citation burst of keywords

**Figure 8:**
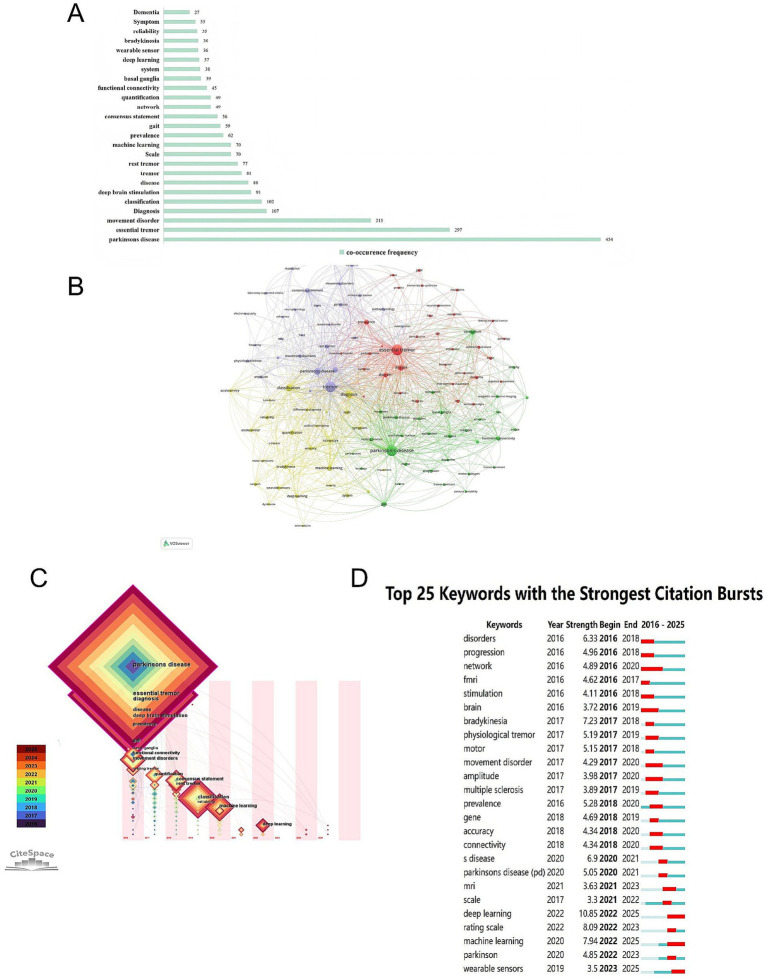
Analysis of keywords. **(A)** Top 25 keywords by co-occurrence frequency. **(B)** Keywords co-occurrence network map. A minimum of 15 co-occurrences were analyzed. Node size is used to visualize total co-occurrence frequency, connecting lines depict co-occurrence strength, and colors differentiate thematic or collaborative clusters. The red cluster focuses on tremor pathophysiology and biomarker discovery. The green cluster highlights neuroimaging and neuroanatomical correlates of tremor. The blue cluster encompasses neurophysiological assessment techniques. The yellow cluster highlights quantitative evaluation methods and digital health technologies. **(C)** Timezone of keywords. Node size represents the total co-occurence strength and colored rings represent the temporal distribution by year This timezone map clearly demonstrates the evolution of keyword related to tremor diagnosis and differentiation during the past decade. **(D)** Top 25 keywords with strongest citation bursts.

Consolidate synonyms and duplicate terms, and select the top 25 high-frequency keywords. As shown in Figure 8A, the two most common keywords were “parkinsonian tremor” and “essential tremor,” which means these two types have dominated the field in the last 10 years. But it also shows that other types, like dystonic tremor and axial tremor, have been largely overlooked. To fill this gap, future studies can focus more on tremor types other than PT and ET.

From a pool of 4,058 keywords, those with a minimum of 15 co-occurrences were analyzed in VOSviewer to construct a co-occurrence network, revealing four thematic clusters (Figure 8B).

Further analysis was conducted using CiteSpace to generate a keyword timezone map (Figure 8C), which illustrates the evolutionary trajectory of the field. Early research focused predominantly on the analysis of tremor-related neurological conditions such as PT and ET, establishing a foundation for subsequent differential diagnosis. Parallel research was directed toward neuroanatomical alterations in tremor, such as “functional connectivity” changes and structural modifications within the “basal ganglia.” Over time, the research focus shifted to tremor classification, which significantly enhanced the precision and refinement of diagnostic and differential capabilities. During the past decade, the field has witnessed growing interest in digital health technologies, with “machine learning” gaining prominence as innovative approaches. This evolution reflects a clear progression in tremor assessment toward objective, quantitative, and remote tremor monitoring.

In addition to keyword clustering and timezone analysis, burst detection mapping (Figure 8D) was used to identify dynamic research fronts and methodologically relevant emerging topics. Burst strength reflects the intensity of increase in keyword co-occurrence over a specific period and therefore indicates rapidly growing scholarly attention (Huang et al., 2025). During 2016–2020, the strongest bursts were mainly associated with neuroanatomical and neuroimaging-related terms, including “fMRI” (strength = 4.62), “brain” (strength = 3.72), and “network” (strength = 4.89), suggesting that early research in this period emphasized brain structure, connectivity, and tremor-related neural mechanisms. In contrast, during the most recent 5 years, methodology-related keywords showed a marked shift toward digital and computational approaches. Specifically, “machine learning” (strength = 7.94), “wearable sensors” (strength = 3.50), and “deep learning” (strength = 10.85) emerged as prominent burst terms, with “deep learning” showing the strongest burst intensity among all detected keywords. These findings indicate that AI-based analysis, sensor-based quantification, and remote digital assessment have become increasingly prominent methodological frontiers in tremor diagnosis and differential diagnosis.

### 3.6 Current status of clinical trials

**Figure 9:**
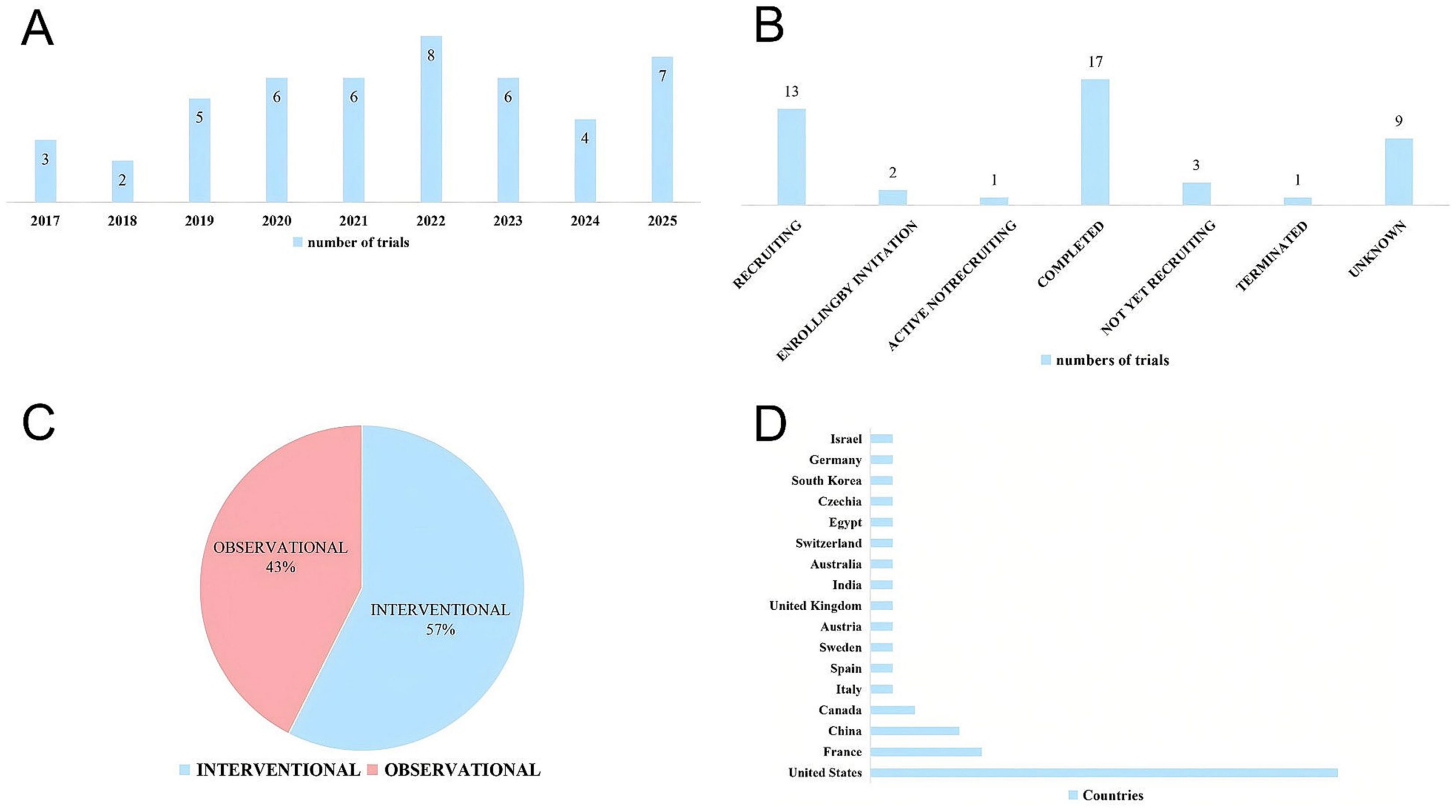
Analysis of clinical trials. **(A)** The number of trials annually. **(B)** The number of trials by study status. **(C)** The ratio of study type. **(D)** The number of trials by countries.

A total of 47 clinical trials focusing on tremor diagnosis and differential diagnosis have been registered over the past decade. The earliest trials were initiated in 2017, with annual numbers increasing steadily and culminating in a peak in 2022, followed by a modest decline during the subsequent 3-year period (Figure 9A). Currently, 16 (34.04%) trials maintain active status, distributed as follows: 13 actively recruiting, 1 active but not recruiting, and 2 enrolling by invitation. Meanwhile, 12 trials have been completed, 3 have not yet begun recruitment, and 1 was terminated (Figure 9B).

As shown in Figure 9C, among these 47 studies, observational research (*n* = 27, 57.45%) slightly outnumbered interventional studies (*n* = 20, 42.55%). The research predominantly focused on ET (*n* = 30, 63.83%), followed by PT (*n* = 20, 42.55%) and dystonic tremor (*n* = 6, 12.77%), indicating that these three tremor types represented the primary subjects of investigation in the field (Table 5). Furthermore, analysis of these data suggests a higher clinical translation rate for ET diagnosis compared to PT.

**Table 5:** Top 3 diseases involved in interventional studies, observational studies, and all studies.

Intervention		Observation		Total	
Conditions	Counts	Conditions	Counts	Conditions	Counts
Essential Tremor	14	Essential tremor	16	Essential Tremor	30
Parkinson Disease	9	Parkinson disease	11	Parkinson Disease	20
Dystonia	5	Progressive supranuclear palsy	3	Dystonia	6

Additionally, interventions utilized in these studies predominantly involved device-based approaches (*n* = 12, 25.53%), including smartphone accelerometer sensors, transcranial ultrasound, electroencephalography (EEG) - functional magnetic resonance imaging (fMRI), and electromagnetic. These were followed by pharmacological interventions (*n* = 7, 14.89%) and diagnostic testing procedures (*n* = 7, 14.89%), such as clinical examination, cerebral magnetic resonance imaging (MRI), optical coherence tomography (OCT), tapping tests, and Archimedes spiral drawing.

Geographically, as shown in Figure 9D, the United States (*n* = 22, 46.81%) accounted for nearly half of all registered trials, substantially exceeding the contributions from France (*n* = 5, 10.64%) and China (*n* = 4, 8.51%). This disparity may be attributed to differences in technological infrastructure, research funding allocation, and capacity for clinical translation. In terms of funding sources, academic medical institutions and research agencies served as the primary funding sources, supporting 33 trials (70.21%). Notably, the University of Texas Southwestern Medical Center, Massachusetts Eye and Ear Infirmary invested in the highest number of trials (*n* = 3, 6.38% each).

## 4 Discussion

### 4.1 Overall research characteristics and future directions

Bibliometric analysis confirms the United States’ dominant position in tremor diagnosis and differentiation research. At both national and institutional levels, the U.S. demonstrates the highest publication output and centrality metrics. In particular, the two most productive institutions are U.S.-based, further reinforcing the country’s preeminent role in this field. At the author level, the most influential individual researcher, Louis, Elan D- a leading authority on tremor diagnosis and differentiation - is also affiliated with a U.S. institution. Additionally, the United States accounted for nearly half of all registered clinical trials over the past decade, highlighting its substantial contribution to clinical translation in this domain.

Regarding collaborative research network, it exhibits decentralized patterns. While this structural characteristic encourages methodological diversity, it simultaneously reveals limited cross-group integration within the field. Moreover, analysis of clinical research activity reveals a limited number of registered trials in this domain, only 47 over the past decade, with merely 12 completed, indicating a relatively low clinical translation rate.

Taken together, these findings suggest that future research should emphasize fostering cross-group, cross-institutional, and international collaborations, as well as enhance innovation and accelerate the clinical translation.

### 4.2 Research hotspots

The co-occurrence network and cluster analysis of keywords (Figure 8B) reveal that current research focuses on two tremor types, PT and ET, and on four major themes: (1) quantitative tremor assessment and digital health technologies, (2) neurophysiological evaluation, (3) neuroimaging, and (4) pathophysiology and biomarkers. This section mainly focuses on these research hotspots mentioned above.

#### 4.2.1 Quantitative tremor evaluation and digital health technologies

The rapid evolution of digital technology and AI has propelled digital health to the forefront of tremor research, as evidenced by the prominence of keywords including “accelerometer,” “wearable sensors,” “artificial intelligence,” “machine learning,” and “telemedicine.”

Accelerometers often serves as the most common integrated sensors in wearable devices (Fu et al., 2024). It can effectively quantify tremor characteristics including frequency and amplitude across three axes, so often surpassing conventional two-dimensional video analysis in specific diagnostic contexts. When integrated with gyroscopes and magnetometers, these sensors constitute inertial measurement units (IMUs) that simultaneously capture three-dimensional linear and angular motion (Sanchez-Perez et al., 2018). Furthermore, IMUs employ correction parameters derived from accelerometer and magnetometer data to compensate for confounding factors such as gravitational effects, thereby significantly improving tremor detection accuracy (Schwingenschuh et al., 2025).

As the second most prevalent embedded sensor for tremor quantification, cameras provide notable portability benefits owing to their widespread availability in both clinical and personal devices (Fu et al., 2024). As a contactless methodology, video analysis minimizes artifacts associated with additional loading (di Biase et al., 2022). Moreover, it can be applied in telemedicine and indoor monitoring, thereby expanding access to professional healthcare services (Angelopoulou et al., 2024). Video-based assessment demonstrates comparable performance to accelerometric approaches; Uhríková et al. reported only 14% of data with frequency discrepancies exceeding 0.2 Hz (Uhríková et al., 2009). Additional investigations have further validated high agreement between video-based assessment and uniaxial accelerometer measurements (Williams et al., 2021).

With the rapid development of digital health technologies, there is an increasing application of AI in tremor research. Machine learning (ML) plays a key role in AI implementation, with DL being a subset of it. Within ML methodologies, support vector machines (SVMs) are the most commonly utilized approach, while convolutional neural networks (CNNs) serve as the most prevalent approach in DL applications (Fu et al., 2024; Vizcarra et al., 2024). Both analytical approaches can be integrated with a range of conventional tremor diagnostic techniques, including radiomics, accelerometry, video analysis, wearable sensors, sEMG, leading to significant transformative impacts (Barrantes et al., 2017; Thorp et al., 2018; Farhani et al., 2022; Tang et al., 2024; Xu et al., 2024). Such integration enhances the precision of feature discrimination and improves the differential diagnosis of various tremor disorders (Barrantes et al., 2017). A recent study utilized a convolutional long short-term memory (LSTM) network combined with wearable accelerometer/gyroscope devices for the monitoring of PT. This approach not only achieved a high diagnostic accuracy of 99.62% but also enabled real-time tremor monitoring, personalized progression analysis, and severity assessment, thereby significantly surpassing the capabilities of conventional diagnostic approaches (Sabari and Ch, 2025). Mahali et al. also employed a deep learning (DL) system to analyze multi-sensor movement data recorded during dynamic motor tasks, successfully differentiating ET, PD, and healthy controls with an overall accuracy of 99.32% (Mahali et al., 2025). Moreover, the integration of DL with handwritten or hand-drawn images allows for accurate remote assessment of tremor, offering promising utility in telemedicine (Anandapadmanabhan et al., 2025; Peng et al., 2025).

#### 4.2.2 Neurophysiological evaluation

Electromyography (EMG) and accelerometry represent the most widely employed neurophysiological techniques in tremor evaluation, as indicated by their high keyword frequencies (van den Berg and Helmich, 2021).

These two methods facilitate the quantification of key tremor characteristics, including peak frequency, frequency variability, burst duration, burst synchrony, and tremor amplitude (Schwingenschuh et al., 2025). EMG serves as a reliable method for detecting pathological tremors by recording the occurrence and intensity of the underlying motor unit entrainment (Haubenberger et al., 2016). Furthermore, Hossen et al. demonstrated that combined accelerometry and surface EMG can effectively differentiate physiological tremor from PT and ET with 93.87% accuracy (Hossen et al., 2020), indicating that the integration of accelerometry and EMG enhances comprehensive tremor evaluation.

Different tremor types possess characteristic electrophysiological and clinical signatures that aid in their differentiation. By providing quantitative characterization of tremor, accelerometry and EMG overcome limitations of purely clinical assessments and substantially improving differential diagnostic accuracy (Schwingenschuh and Espay, 2022).

Parkinson’s tremor typically manifests asymmetrically, primarily involving the limbs or jaw (Levi-Strauss et al., 2025), and is characterized by high amplitude and a 4–7 Hz resting tremor with a “pill-rolling” appearance (Williams et al., 2021). This tremor tends to worsen under cognitive load or during contralateral motor tasks, demonstrates suppression during voluntary movement, and may re-emerge following a delay of several seconds to minutes (re-emergent tremor) (Schwingenschuh et al., 2025).

Essential tremor typically manifests with a frequency of 7–11 Hz and lower amplitude compared to PT (Chen and Chen, 2020). Gironell et al. established six neurophysiological diagnostic criteria for ET:(1) rhythmic bursts of postural tremor on sEMG, (2) tremor frequency≥4 Hz; (3) absence of rest tremor; (4) absence of latency from rest to postural position, (5) changes in the dominant frequency peak≤1 Hz after loading, and (6) no changes in tremor amplitude during cognitive load. Fulfillment of all six criteria achieves 97.7% sensitivity and 82.3% specificity for ET diagnosis (Deuschl et al., 2022).

Given the numerous distinct neurophysiological features between PT and ET, multiple features can be employed to differentiate the two disorders: (1) High EMG amplitudes and short burst durations suggest PD, whereas a long burst duration and low tremor amplitude favor an ET diagnosis (Chen and Chen, 2020); (2) PD shows alternating contractions of agonist–antagonist muscle, in contrast to the synchronous pattern of ET; (3) Cognitive load increases tremor amplitude in PD but does not affect ET; (4) Patients with PD display a significantly delayed onset of postural tremor compared to those with ET or enhanced physiological tremor (Zhang et al., 2017); (5) The mean harmonic peak power (MHP) is higher in PD than in ET, a feature that achieves 91.7–94% accuracy in distinguishing PD from ET (van der Veen et al., 2021); (6) Frequency variability is lower in PT than in ET. Among those measured parameters, the Tremor Stability Index (TSI), defined as the interquartile range of tremor frequency variation measured by accelerometry, contributed most significantly to tremor variation (di Biase et al., 2017; van der Veen et al., 2021). This feature outperformed the mean harmonic peak power (MHP) in distinguishing PT from ET in most experimental time, with 95% sensitivity and 95% specificity (di Biase et al., 2017). Building on this, Luft et al. (2019) introduced the concept of tremor windows (TWs) and employed 3D accelerometry combined with surface EMG to differentiate TWs from non-TW tremors (NTWs). Their findings indicate that analyzing TWs not only facilitates TSI estimation but also improves the discrimination between PT and ET. Additionally, Mahali et al. (2025) achieved 99.32% accuracy in differentiating PT from ET using accelerometry combined with transformation models, surpassing previous findings. In parallel, IMUs have also shown high precision in classifying atypical tremor, PT, ET, psychogenic tremor, slow orthostatic tremor, and primary orthostatic tremor (Onder et al., 2025).

In terms of dystonia, two primary tremor phenotypes are recognized in it: dystonic tremor (DT) and tremor associated with dystonia (TAWD). DT often manifests as irregular, jerky tremors manifesting as rhythmic bursts that last 50–300 ms (van der Veen et al., 2021). Compared to ET, DT exhibits a higher tremor stability index (TSI), indicating greater variability in frequency (Shaikh et al., 2008; van der Veen et al., 2021). In contrast, TAWD typically presents with a regular, sinusoidal tremor (Fahn, 1984).

The abovementioned tremor types belong to the category of organic tremors, which require differentiation from functional tremors. Functional tremors exhibit distinctive neurophysiological profiles from organic tremors, most notably positive electrophysiological signs including distractibility and entrainment (Schwingenschuh and Espay, 2022). In functional tremor, voluntary rhythmic movements of the unaffected limb may produce either entrainment, characterized by significant coherency between the EMG spectra of the tremulous hand and that of the moving extremity, or complete tremor suppression (Deuschl et al., 2022). Conversely, during distraction or ballistic movements of the unaffected extremity, tremor amplitude may decrease or cease transiently, a finding demonstrating high specificity for functional tremor diagnosis (Chen and Chen, 2020). Additionally, functional tremor is frequently preceded by approximately 300 ms of co-activation in the agonist and antagonist muscle. In such cases, slow passive movement can reduce this co-activation and lead to tremor cessation. Furthermore, amplitude increase during mass loading (Deuschl et al., 2022), which has high specificity (92%) but low sensitivity (22%) for functional tremor diagnosis (Chen and Chen, 2020). This indicates that while not all functional tremors exhibit this sign, its presence provides strong evidence for a functional etiology. When multiple body regions are affected by functional tremors, high coherence of tremor frequencies across sites is typically observed. Moreover, the neurophysiological profile is further characterized by the reduced number of periods without significant coherence (NOV) between flexor and extensor muscles (Kramer et al., 2018). Recent studies, such as those by Schwingenschuh et al., have employed a set of electrophysiological features as diagnostic criteria for functional tremor, such as co-activation patterns, response to mass loading, tremor frequency modulation during contralateral tapping, task-dependent tremor variability, bilateral coherence, and tremor suppression following ballistic movements. A composite scoring system, in which a score of three or more points out of 10 supports the diagnosis, has demonstrated a sensitivity of 89.5%, a specificity of 95.9%, and high inter-rater reliability (Schwingenschuh et al., 2016). However, diagnostic accuracy can be compromised when functional and organic tremor coexist, as phenomena such as tremor entrainment and mirror movements are often confused (Merchant et al., 2018). Future research should therefore focus on the effectiveness of using these electrophysiological signs to diagnose functional tremor that is masked by organic tremor.

#### 4.2.3 Neuroimaging evaluation

Neuroimaging techniques can reveal tremor-related changes in brain structure, functional activity, and connectivity networks. As indicated by keywords in green cluster highlighting neuroimaging and neuroanatomical (Figure 8B), such as “basal ganglia,” “cerebellum,” “cortex,” and “subthalamic nucleus,” structural alternations within these regions are closely linked to the pathogenesis of tremor. Meanwhile, “magnetic resonance imaging” and “voxel-based morphometry” are two of the most widely used neuroimaging techniques for evaluating such structural and functional brain changes in tremor. These techniques can be classified into several categories: structural neuroimaging, including voxel-based morphometry, diffusion-weighted imaging (DWI), diffusion tensor imaging (DTI); functional neuroimaging, such as positron emission tomography (PET), single-photon emission computed tomography (SPECT), fMRI; and magnetic resonance spectroscopy (MRS). Each method possesses different strengths and limitations. When combined in a multimodal approach, they enable a comprehensive assessment of cerebral structure, functional dynamics, and metabolic activity.

In terms of the neuroimaging evaluation for PT, evidence indicates that PT is triggered in the globus pallidus internus (GPi) of the basal ganglia (Prasad et al., 2024), relayed through the motor cortex (M1), and amplified within the cerebello-thalamo-cortical circuit (Helmich et al., 2011; Dirkx et al., 2016; van den Berg and Helmich, 2021; Prasad et al., 2024). Within this circuit, cerebellum conveys excitatory signals from the deep cerebellar nuclei (DCN) to the ventral intermediate nucleus (VIM) of the thalamus, thereby setting the cerebral cortex into a feedforward signal processing mode (Buijink et al., 2022). This mechanism, in the tremor state, supports the propagation and amplification of tremor-associated oscillations.

Essential tremor also involves the cerebello-thalamo-cortical network (Dirkx et al., 2019). Head and neck tremor is associated with atrophy in the midline regions of the anterior cerebellar lobe, particularly involving the vermis and lobules IV–V (Handforth, 2016). In cases of hand and leg tremor, atrophy involves in both the anterior lobe and the cerebellar hemispheres. This process may be related to regional iron deposition (Novellino et al., 2013). Such cerebellar neurodegeneration leads to Purkinje cell loss and decreased GABA receptor expression in the dentate nucleus (DN) (Handforth, 2016; Pan and Kuo, 2022), ultimately resulting in cerebello-thalamo-cortical circuit hyperactivity and tremor generation. In addition, evidence finds that the inferior olive is also implicated in the pathogenesis of ET. Climbing fibers originating from this structure extend distally to innervate Purkinje cells in the cerebellar cortex. Neuronal synchrony in the inferior olive is regulated by GABAergic inputs from the DCN—notably the DN —forming a functionally integrated olive-cerebellar-cortical loop (Pan and Kuo, 2022). Deficits in GABAergic inhibition within the DN enhance synchronized firing among inferior olive neurons, inducing oscillatory activity that contributes to tremor manifestation. Therefore, degeneration of cortical Purkinje cells compromises their inhibitory terminals in the olive, leading to PC terminal axonal sprouting in the DCN that innervates larger populations of olivary neurons. This maladaptive rewiring results in excessive recruitment of PC complex spikes, ultimately initiating tremor (Handforth, 2016).

Nevertheless, establishing causal links between cerebral alterations and tremor etiology remains challenging through neuroimaging alone. As reviewed by van den Berg and Helmich, several methodologies have been employed to address this limitation, including combined EMG-neuroimaging, dynamic causal modeling (DCM), directed coherence analysis of EEG/MEG signals, and interventions combined with neuroimaging (van den Berg and Helmich, 2021). Additionally, currently neuroimaging research in tremor predominantly focuses on mechanism rather than clinically diagnostic. Therefore, future research should focus more on the aforementioned two aspects, broadening the scope of tremor neuroimaging and enhancing its clinical applicability.

As neuroimaging techniques can characterize the structural and functional features of the brain across different tremor phenotypes, they are widely employed in the classification of tremor. In this context, rs-fMRI stands as one of the most extensively utilized neuroimaging approaches. X. Zhao et al. utilized rs-fMRI to differentiate between tremor-dominant multiple system atrophy (MSA) and tPD. The result revealed increased mean percent amplitude of fluctuation (mPerAF) values and enhanced effective connectivity in the bilateral putamen in tremor-dominant MSA, whereas both metrics were reduced in tPD (Li et al., 2025). These findings confirm the putamen as a key neural marker for distinguishing tremor phenotypes in MSA and PD. In a recent study, a multimodal connectivity graph convolutional network (MGGCN) applied to rs-fMRI data achieved 88.8% accuracy in differentiating rET and tPD. Regions most implicated in this discrimination included the left cerebellum, the right basal ganglia (within the cingulo-opercular network), and the supplementary motor area (SMA). The study also found that the occipital network and the basal ganglia-temporal lobe network may serve as tremor-related networks for rET and tPD, respectively (Zhao et al., 2024). However, the mechanistic basis of these network-specific contributions requires further investigation.

Dopamine transporter single-photon emission computed tomography (DaT-SPECT) is also a commonly used neuroimaging method in tremor classification. It can accurately assess the degeneration of nigrostriatal dopaminergic neurons in PD, thereby providing a reliable means of distinguishing PT from other tremor disorders, such as ET (Salsone et al., 2016). Additionally, Transcranial Sonography (TCS) is another technique that aids in differentiating PT from other tremor types by assessing alterations in the substantia nigra (SN) and striatum. A transcranial sonography (TCS) study of 500 patients at Beijing Tiantan Hospital revealed a significantly higher substantia nigra (SN) positive rate in the PD group compared to the ET, MSA, and PSP groups (The ratio of the bilateral SN hyperechoic area to the total midbrain area (S/M) ≥ 7% and/or an absolute SN area ≥ 0.25 cm^2^ indicated SN to be positive). And the lenticular nucleus (LN) (i.e., the striatum) hyperechoic area was larger in the PD group than in the ET and MSA groups, but smaller than that in the PSP group (Wang et al., 2021). A recent meta-analysis evaluating transcranial sonography (TCS) for the differential diagnosis of ET and PD reported pooled sensitivity and specificity of 84.6 and 83.9%, respectively, based on substantia nigra hyperechogenicity. The study also demonstrated that TCS has sensitivity and specificity comparable to DaTSCAN in distinguishing PD from ET (Heim et al., 2022).

Other imaging techniques used less frequently in clinical practice—such as MRI relaxometry (Filip et al., 2020), nigrosome-1 imaging (Wang et al., 2019), susceptibility map-weighted imaging (SMWI)(Jo et al., 2025), heart rate variability (HRV), and [^123^I]MIBG cardiac scintigraphy—also provide useful information for differentiating PD from ET (Salsone et al., 2016).

#### 4.2.4 Pathophysiology and biomarkers of tremor

Tremor onset is commonly linked to multiple pathophysiological changes, such as abnormal cerebral metabolism. These pathological changes are detectable in biofluids such as blood and cerebrospinal fluid (CSF), manifested as changes in specific biomarkers, notably *α*-synuclein. Alpha-synuclein, a misfolded and aggregated protein in the brain, is among the most extensively studied biomarkers in PD (Ma et al., 2024). Decreased levels of total α-synuclein have been found in the CSF of PD patients and in the serum of ET patients, respectively, whereas serum total α-synuclein, neuronally derived extracellular vesicle (NDEV) oligomeric and p129-α-synuclein, erythrocyte α-synuclein levels in PD and ET cohorts are significantly elevated compared to healthy controls (Ma et al., 2024; Shalash et al., 2024; Tsao et al., 2022; Yu et al., 2023; Cristiani et al., 2025). Several α-synuclein-related biomarkers have demonstrated high diagnostic accuracy, including elevated salivary α-synuclein seeding activity, salivary miRNA-29a-3p levels, oligomeric α-synuclein in NDEVs, and an increased oligomeric-to-monomeric α-synuclein ratio in erythrocytes (Yu et al., 2023; Luan et al., 2024; Cristiani et al., 2025). Among these, NDEV oligomeric α-synuclein achieved the highest accuracy in differentiating PD and ET at 99.7% (Cristiani et al., 2025). A gas chromatography–mass spectrometry-based study also identified significantly elevated levels of plasma and CSF α-synuclein, plasma phosphorylated Ser129 α-synuclein (pSer129α-syn), and the ratio of plasma pSer129α-syn/total α-syn in PD patients compared to ET (Ostrakhovitch et al., 2022). The research further shows that the oxidative branch of the pentose phosphate pathway (PPP) shows increased activity in PD, with suppression of the non-oxidative branch. This metabolic shift leads to elevated plasma levels of NADPH and NADP+, along with decreased ribose. NAMPT activates pro-inflammatory pathways and may contribute to ischemic necrosis of neurons in PD patients (Zhao et al., 2013; Audrito et al., 2020; Ostrakhovitch et al., 2022).

Proton magnetic resonance spectroscopy (^1^H-MRS) is also a commonly used technique for detecting molecular and metabolic changes associated with tremor. It is capable of quantifying changes in metabolite concentrations within tissues, offering considerable clinical value. Findings from ^1^H-MRS have revealed an elevated GABA+/Cr ratio in the cerebellum of patients with ET and DT, potentially indicating enhanced inhibitory interneuron activity, such as basket and stellate cells. It is worth noting that, despite Purkinje cells being major GABAergic neurons, their reduction in the cerebellum of ET patients suggests that the GABA increase is not attributable to these cells (Bedard et al., 2022). Further investigations have reported elevated Glx concentrations and Glx/Cr ratios in the thalamus of ET patients, while NAA/Cr and Ch/Cr ratios remain unaltered. These findings potentially point to thalamic hyperactivity without neuronal loss or membrane injury, supporting the notion that functional—rather than structural—changes underlie ET thalamic pathology (Barbagallo et al., 2018). In contrast to ET, PD patients demonstrate notably decreased GABA+ levels in the left basal ganglia (all subjects are right-handed), with significantly lower concentrations observed in the tPD than in the PIGD subtype (Gong et al., 2018). Moreover, thalamic NAA/Cr and Ch/Cr ratios are reduced in tPD, suggesting neuronal loss and impaired membrane integrity, which may be associated with alpha-synuclein pathology and dysregulated cholesterol metabolism in the thalamus (Barbagallo et al., 2017). Together, these findings display distinct neurochemical profiles between tPD and ET, particularly in GABA+, NAA/Cr, and Ch/Cr. Notably, the combination of NAA/Cr and Ch/Cr ratios has achieved 100% accuracy in differentiating tPD from essential tremor with resting tremor (rET) and healthy controls, whereas the utility of GABA+/Cr for distinguishing tPD from ET necessitates further research.

### 4.3 Research frontiers

Bibliometric analysis involving keyword clustering, keyword timezone and burst detection systematically reveals the field’s evolutionary trajectory and emerging frontiers. Prior to 2016, research predominantly focused on PT and ET. During the subsequent period (2016–2020), research focus shifted toward neuroanatomical correlates of tremor, which emerged as a frontier hotspot. Since 2019, tremor classification has gained prominence. Most recently, digital health technologies, particularly in the domains of “deep learning” and “machine learning,” have accelerated rapidly over the past decade, establishing these as critical future research directions.

ML and DL are increasingly integrated into tremor diagnosis and differential diagnosis, with considerable progress already demonstrated. However, contemporary ML applications face specific limitations. Firstly, current research is heavily focused on model development, with insufficient attention to subsequent validation and real-world clinical practice. This gap significantly constrains the demonstrated clinical effectiveness of ML/DL–based tools (De et al., 2023). Secondly, a dependence on established features that constrains the scope of data exploration. Although DL models can overcome this by automating feature extraction, they demand extensive training datasets and substantial computational resources. Thirdly, as population characteristics, data quality, and clinical management standards evolve, ML/DL models require continuous external monitoring to maintain diagnostic validity. Moreover, ML/DL models are often regarded as “black boxes” due to the complex internal decision-making processes that are difficult to disentangle. This lack of transparency undermines their perceived reliability and clinical acceptability, thereby substantially limiting the adoption and translation of DL in tremor diagnostics (Sánchez-Fernández, 2024).

In the near future, ML/DL will undoubtedly play an increasingly larger role in medicine, and the above-mentioned limitations of this digital health technology should be carefully monitored by researchers in this field.

## 5 Limitations

This study is subject to several methodological limitations. Firstly, the literature search was confined to English-language publications indexed in the Web of Science Core Collection (WOSCC) and to trials registered on ClinicalTrials.gov, which may introduce database coverage bias. Compared with Google Scholar, Embase, PubMed, and Scopus, WOSCC covers fewer records, and institutional access to WOS varies (Falagas et al., 2008; Gusenbauer, 2022). WOSCC also provides lower coverage in physics, computer science, and engineering than Scopus (Gusenbauer, 2022). This is a relevant limitation because our research involves AI, wearables, and engineering methods that overlap with those fields.

Additionally, the scope covers literature from 2016 to August 31, 2025, resulting in a lack of comprehensive analysis of earlier and more recent publications, and an incomplete evaluation of publication trends approaching 2025. Furthermore, our Discussion section focuses primarily on PT and ET, as they reflect the actual research activity captured in our bibliometric analysis, with some coverage of dystonic tremor (DT) and functional tremor. However, we have largely overlooked many other tremor types, such as axial tremor. Moreover, as PT and ET accounted for the overwhelming majority of publications in our dataset, our bibliometric analysis primarily reflects research trends in these two phenotype. Consequently, the overall trends and hotspots identified in this study are largely representative of these two phenotypes, while other tremor types, such as dystonic tremor and axial tremors, were relatively under-represented. This imbalance limits the generalizability of our findings to the full spectrum of tremor disorders.
